# Enhypyrazinones A and B, Pyrazinone Natural Products from a Marine-Derived Myxobacterium *Enhygromyxa* sp.

**DOI:** 10.3390/md17120698

**Published:** 2019-12-12

**Authors:** Fan Zhang, Doug R. Braun, Scott R. Rajski, Don DeMaria, Tim S. Bugni

**Affiliations:** 1 Pharmaceutical Sciences Division, University of Wisconsin–Madison, Madison, WI 53705, USA; 2 Sea Samples, 369 Westshore Drive, Summerland Key, FL 33042, USA

**Keywords:** pyrazinone, marine-derived myxobacterium, *Enhygromyxa* sp.

## Abstract

To date, studies describing myxobacterial secondary metabolites have been relatively scarce in comparison to those addressing actinobacterial secondary metabolites. This realization suggests the immense potential of myxobacteria as an intriguing source of secondary metabolites with unusual structural features and a wide array of biological activities. Marine-derived myxobacteria are especially attractive due to their unique biosynthetic gene clusters, although they are more difficult to handle than terrestrial myxobacteria. Here, we report the discovery of two new pyrazinone-type molecules, enhypyrazinones A and B, from a marine-derived myxobacterium *Enhygromyxa* sp. Their structures were elucidated by HRESIMS and comprehensive NMR data analyses. Compounds **1** and **2**, which contain a rare trisubstituted-pyrazinone core, represent a unique class of molecules from *Enhygromyxa* sp.

## 1. Introduction

**Figure 1:**
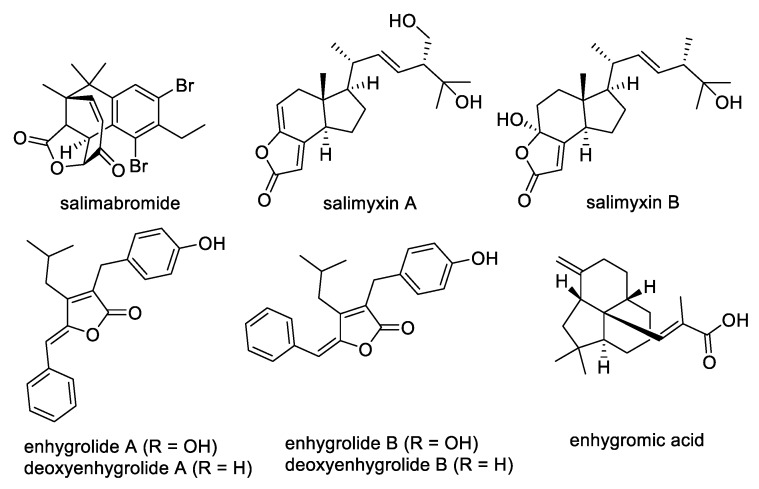
Secondary metabolites from *Enhygromyxa* spp.

Myxobacteria are Gram-negative gliding bacteria with large genomes; these organisms also undergo complex multicellular developmental processes that lead to fruiting body formation [1]. Most myxobacteria have a heterotrophic lifestyle and feed on different bacteria and fungi through assorted predatory behaviors [2]. Compared to their non-predatory relatives, predatory myxobacteria possess a higher density of secondary metabolite gene clusters in their genomes; this suggests their great promise as repositories for the discovery of novel natural products [3]. Terrestrial myxobacteria usually do not tolerate NaCl concentrations greater than 1.0%, and before 2005 the vast majority of characterized myxobacteria were obtained from terrestrial habitats. Recently, however, four new genera of halotolerant and obligate marine myxobacteria, *Enhygromyxa*, *Haliangium*, *Plesiocystis*, and *Pseudenhygromyxa*, have been discovered and classified [4,5,6,7,8,9]. Although not extensively studied, the limited records of marine-derived myxobacteria indicate their immense potential as prolific producers of novel natural compounds with prominent biological activities, making these social microbes highly attractive for drug discovery [10,11,12,13,14,15,16]. The genus *Enhygromyxa*, in particular, has been shown to produce novel bioactive molecules, including salimabromide [17], salimyxins A and B [18], enhygrolides A and B [18], enhygromic acid [19], and deoxyenhygrolides A and B (Figure 1) [19].

**Figure 2:**
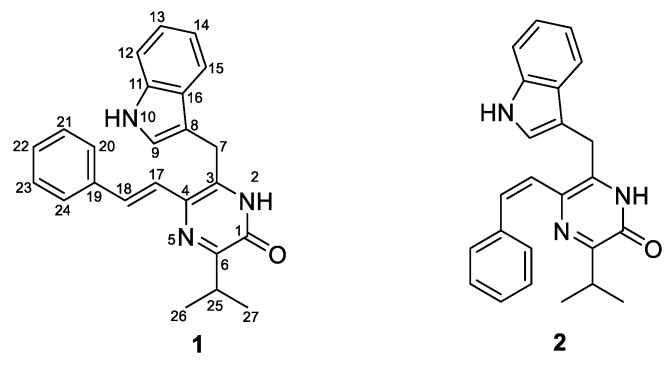
Two new pyrazinone derivatives: enhypyrazinones A (**1**) and B (**2**).

In our efforts to explore the potential of myxobacteria as a source of unique unknown natural products, we isolated a marine myxobacterium from the sponge *Biemna* sp., which was identified as *Enhygromyxa* sp. WMMC2659 by 16S sequence analysis. Metabolomics-guided fractionation of strain WMMC2695 led to the discovery of two new pyrazinone derivatives, termed here as enhypyrazinones A (**1**) and B (**2**) in Figure 2.

## 2. Results

### 2.1. Cultivation Conditions of Marine-Derived Myxobacterium Enhygromyxa sp. WMMC2695

**Figure 3:**
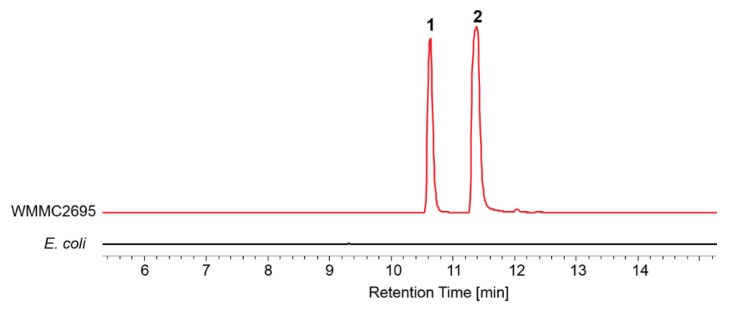
Extracted ion chromatogram (EIC) traces (*m*/*z* 392) of the culture extracts from WMMC2659 (living *Escherichia coli* DH5α as media) and *E. coli* DH5α.

Marine myxobacteria are particularly rare and difficult to handle, because their nutritional and metabolic growth requirements are in most cases not well understood, and need to be determined on a case-by-case basis for each strain. Until now, only a few isolates could be cultured under laboratory conditions. Unlike faster-growing microbes, nutrient-lean media are preferable for marine myxobacteria as they enable the germination of myxospores, and later support swarming of the vegetative cells [20]. Based on previous reports about conditions for isolation and cultivation of *Enhygromyxa* spp., *Escherichia coli* [17,18], half-strength yeast cell VB12 medium (VY/2) [4,19], half-strength VY/2 (VY/4) [17,19], and one-third-strength casitone yeast extract medium (1/3 CY) [4] can be used to provide nutrition for the growth of *Enhygromyxa* spp. Therefore, several media conditions including living *E. coli*, dead *E. coli*, VY/2, VY/4, CY, 1/3 CY, and 1/6 CY were tested for the growth of WMMC2659. R2A agar was also included as one of the testing media due to our experiences with other halotolerant myxobacterial isolates. WMMC2659 was found to be cultivable only on living and dead *E. coli* DH5α, while casitone-containing media seemed to inhibit the growth of WMMC2659 (Table 1). Furthermore, the growth of WMMC2659 on autoclaved *E. coli* DH5α was substantially impaired compared to similar systems employing live cells. More importantly, comparisons of the LC-MS analyses of the culture extract from WMMC2659 (living *E. coli* DH5α as media) and that of *E. coli* DH5α (Figure 3) clearly indicated that WMMC2659 produced two metabolites when using *E. coli* DH5α as nutrition.

### 2.2. Structure Elucidation of Enhypyrazinones A and B

**Figure 4:**
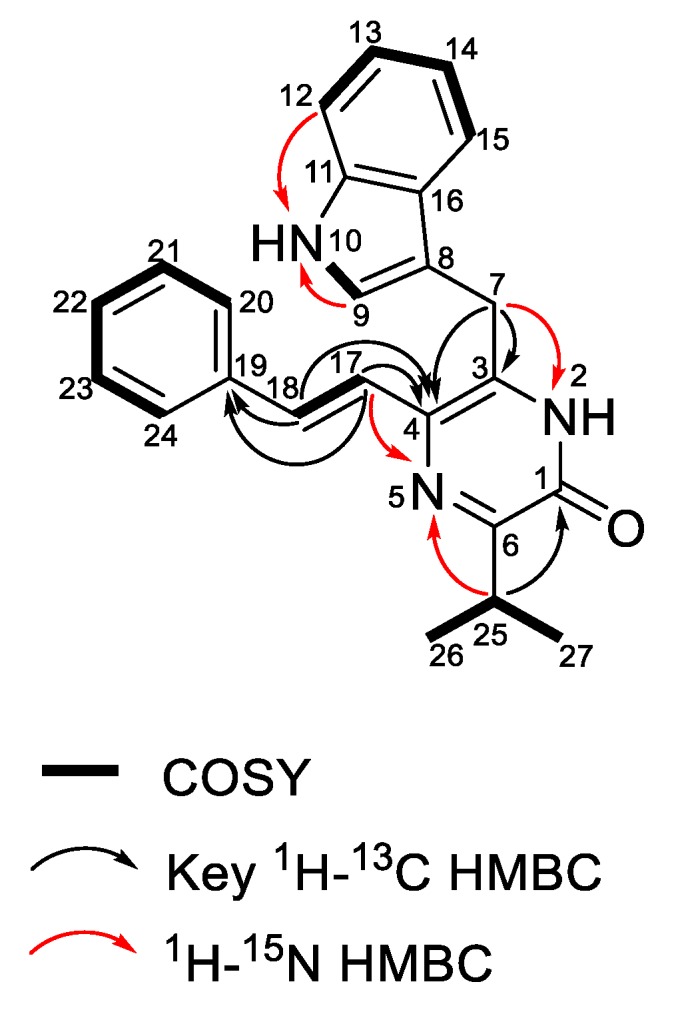
Key 2D NMR correlations for compound **1**.

The molecular formula of enhypyrazinone A (**1**) was determined to be C_24_H_23_N_3_O on the basis of the HRESIMS data ([M + Na]^+^, *m*/*z* 392.1736), indicating 15 degrees of unsaturation. Analysis of the ^1^H and ^13^C NMR and HSQC spectra (Table 2) revealed the presence of eight non-protonated sp^2^-type carbons, 12 olefin/aromatic protons, one methine, one methylene, and two methyl groups. The presence of five aromatic protons at *δ*_H_ 7.51 (H-20/24), 7.32 (H-21/23) and 7.20 (H-22), together with COSY correlations among these aromatic protons (Figure 4), suggested a mono-substituted benzene ring. The HMBC correlations from H-17 and H-18 to C-19 suggested that an olefin was attached to the mono-substituted benzene ring via C-19. Additionally, COSY and ^1^H–^13^C HMBC spectra of **1** revealed a typical 3-substituted indole alkaloid moiety [21] with signals at *δ*_H_ 7.22 (H-9), 11.0 (NH-10), 7.33 (H-12), 7.05 (H-13), 6.97 (H-14), 7.65 (H-15), and *δ*_C_ 111.1 (C-8), 123.6 (C-9), 136.2 (C-11), 111.6 (C-12), 121.1 (C-13), 118.5 (C-14), 118.6 (C-15), 126.7 ppm (C-16). Additionally, HMBC correlations from H_2_-7 to C-8, C-9, and C-16 suggested linkage of the methylene to the indole moiety via C-8. In addition, HMBC correlations from H-17 and H-18 to C-4, and from H_2_-7 to C-3, indicated the linkages between C-17 and C-4, and between C-7 and C-3, respectively. The COSY correlations between the two methyl groups (H_3_-26 and H_3_-27) and the methine (H-25) established the isopropyl spin system, and HMBC correlations from H-25, H_3_-26, and H_3_-27 to C-6 located the isopropyl group at C-6. Although three substructures were deduced, four non-protonated carbons (C-1, C-3, C-4, and C-6), two nitrogens, and one oxygen remained unaccounted for. Therefore, the ^1^H–^15^N HMBC spectrum was used to unambiguously complete the elucidation of compound **1**. The cross-peaks for H-25 and H-17 with N-5 indicated that the linkage between C-4 and C-6 involved N-5 (Figure 4). The HMBC correlations from H_2_-7 to C-4 and N-2 revealed that C-3 was attached to C-4 and N-2. An additional HMBC correlation from H-25 to C-1 indicated the connection between C-6 and C-1. Lastly, the carbonyl carbon C-1 (*δ*_C_ 156.3) was attached to N-2 to form an α,β-unsaturated amide, thus illustrating the presence of a pyrazinone core. The remaining exchangeable proton was assigned as 2-NH to satisfy the molecular formula. On the basis of the large vicinal ^1^H−^1^H coupling constants (*J* = 15.5 Hz), the alkene (H-17, H-18) configuration was assigned as *E* [22,23]. Taken together, the structure of **1** was therefore established.

Enhypyrazinone B (**2**) was found to have the same molecular formula (C_24_H_23_N_3_O) as **1** on the basis of HRESIMS data. Unfortunately, DMSO-*d*_6_ was a poor NMR solvent for compound **2** (Figures S14–S19), and thus a different NMR solvent system (CDCl_3_/CD_3_OD 1:1) was used for NMR studies of **2** (Figures S8–S12). Interpretation of 1D and 2D NMR data sets suggested that **2** is a stereoisomer of **1**. A relatively small coupling constant (*J* = 12 Hz) between H-17 and H-18 indicated that the geometry of the alkene (H17, H18) was *Z* [22,23], thereby enabling unambiguous assignment of the structure for **2**. All other data supported this conclusion. 

### 2.3. Bioactivity Testing

Compounds **1** and **2** were tested for antibacterial activity against *E. coli* (ATCC 25922), methicillin-resistant *Staphylococcus aureus* (MRSA; ATCC 33591), and methicillin-sensitive *Staphylococcus aureus* (MSSA; ATCC 29213) in disk diffusion assays; only weak activity against MSSA was noted. To gain more accurate antimicrobial bioactivity data for compounds **1** and **2**, we determined minimum inhibitory concentration (MIC) values for each species against MSSA. Notably, both **1** and **2** were characterized by MIC values >128 μg/mL, consistent with very low antibacterial activities. Compounds **1** and **2** do not appear to exert antimicrobial effects that clearly benefit the producer, since *E. coli* DH5α was used to provide nutrition for WMMC2659. Moreover, **1** and **2** are more easily and economically biosynthesized than larger signaling peptides, and thus might have a function related to myxobacterial intra- and extra-species cell-cell interaction.

## 3. Discussion

In conclusion, two pyrazinone-type metabolites, **1** and **2**, were isolated from a marine-derived myxobacterium *Enhygromyxa* sp. Notably, this is the first report of natural products generated from this bacterial genus under fermentation conditions involving myxobacterial predation and feeding on a different bacterium (*E. coli* DH5α). Compounds **1** and **2** contain a rare trisubstituted pyrazinone core, and only a few metabolites bearing this moiety have been reported so far. The most related pyrazinone derivatives are tyrvalin and phevalin, from a pathogenic *Staphylococcus aureus* strain [24,25]; leuvalin, which was found in pathogenic strains of *S. aureus*, *S. epidermidis*, *S. capitis*, and *S. lugdunensis* [26]; arglecin, argvalin, JBIR-56, and JBIR-57 from *Streptomyces* sp. [27,28]; sorazinone B, which was from *Nannocystis pusilla* strain MNa10913 [29]; as well as butrepyrazinone, from a Ghanaian *Verrucosispora* sp. K51G [30]. In most cases, the C-4 of the pyrazinone ring is unsubstituted. It appears that the pyrazinones are generated by multidomain non-ribosomal peptide synthetase (NRPS) assembly line systems. Notably, for the systems examined thus far, it appears that cleavage from the NRPS proceeds via thioester reduction; this affords a *C*-terminal aldehyde upon which the *N*-terminal amino group condenses to afford an imine; ultimately, oxidation of this newly formed heterocycle affords 2(1H)-pyrazinones [25]. For previously reported pyrazinone-producing microbes, the typical absence of a substituent at C-4 is logical given its aldehydic oxidation state following dipeptide liberation from the NRPS machinery [24,25,26,27,28,29,30]. In the case of **1** and **2**, it is presently unclear precisely how C-4 comes to be the attachment point for the cinnamoyl moiety, although this question is currently under investigation.

## 4. Materials and Methods

### 4.1. General Experimental Procedures

UV spectra were recorded on an Aminco/OLIS UV-Vis Spectrophotometer (Bogart, GA, USA). IR spectra were measured with a Bruker Equinox 55/S FT–IR Spectrophotometer (Santa Barbara, CA, USA). Both 1D and 2D NMR spectra were obtained using a Bruker Avance 500 MHz spectrometer (Billerica, MA, USA) with ^1^H{^13^C/^15^N} cryoprobe and a 500 MHz spectrometer with ^13^C/^15^N{^1^H} cryoprobe; chemical shifts were referenced to the residual solvent peaks (CD_3_OD: *δ*_H_ = 3.31, *δ*_C_ = 49.15; DMSO-*d_6_*: *δ*_H_ = 2.50, *δ*_C_ = 39.51). HRMS data were acquired with a Bruker MaXis 4G QTOF mass spectrometer (Billerica, MA, USA). RP HPLC was performed using a Shimadzu Prominence HPLC system and a Phenomenex Luna C_18_ column (250 × 10 mm, 5 µm) (Torrance, CA, USA).

### 4.2. Biological Material

WMMC2659 was isolated from the sponge *Biemna* sp. which was collected in the Florida Keys, USA (24 39.393, −81 26.268) on August 13th, 2014. A voucher specimen of the sponge is housed at the University of Wisconsin-Madison. WMMC2659 was isolated using the baiting technique as described by Iizuka et al. [5e], only ground sponge was used in place of soil. WMMC2659 was maintained at 28 °C on plates containing 50% artificial seawater (ASW) with 1.5% agar streaked with *E. coli* DH5α on the surface as the food source. Artificial seawater solutions I (415.2 g NaCl, 69.54 g Na_2_SO_4_, 11.74 g KCl, 3.40 g NaHCO_3_, 1.7 g KBr, 0.45 g H_3_BO_3_, 0.054 g NaF) and II (187.9 g MgCl_2_·6H_2_O, 22.72 g CaCl_2_·2H_2_O, 0.428 g SrCl_2_·6H_2_O) were made up separately and combined to give a total volume of 20 L.

### 4.3. Sequencing

The 16S rRNA gene was amplified using colony PCR with the primers 8–27F (5’ to 3’ GAGTTTGATCCTGGCTCAG) and 1492R (5’ to 3’ GGTTACCTTGTTACGACTT). The following PCR conditions were used: 94 °C for 5 min, followed by 35 cycles of 94 °C for 30 s, 55 °C for 1 min, 72 °C for 1.5 min, with a final step of 72 °C for 5 min. The PCR bands were excised from the gel and purified using the QIAquick Gel Extraction kit (QIAGEN, Germantown, MD, USA). Sanger sequencing was performed at the UW Biotechnology Center. WMMC2695 was identified as an *Enhygromyxa* sp. The 16S sequence for WMMC2695 was deposited in GenBank (accession number MN657412).

### 4.4. Fermentation, Extraction, and Isolation

A starter culture was prepared by scraping fruiting bodies off of a petri dish and inoculating them into a 125 mL Erlenmeyer flask containing ~1−2 g of washed living *E. coli* DH5α in 25 mL 50% ASW buffered with HEPES (25 mM, pH 7.8). When the culture showed orange coloration, it was inoculated into 1 L of ASW containing 30−35 g living *E. coli* DH5α, cyanocobalamin (0.5 mg), Diaion HP20 (7% by weight) and buffered with HEPES (25 mM, pH 7.8). The culture was shaken at 200 rpm at 28 °C for 9 days before extraction. Living *E. coli* DH5α was prepared by growing overnight cultures in 2xTY and washing them 3 times with sterile MilliQ water. Filtered HP20 was washed with distilled H_2_O and extracted with acetone. The acetone extract (2.0 g) was subjected to liquid–liquid partitioning using 30% aqueous CH_3_OH and CHCl_3_ (1:1). The CHCl_3_-soluble partition (0.2 g) was fractionated by Sephadex LH20 column chromatography (CHCl_3_:CH_3_OH, 1:1). The fractions containing **1** and **2** were further subjected to RP HPLC (45%/55% to 90%/10% CH_3_OH-H_2_O over 26 min, 4.0 mg/mL) using a Phenomenex Luna C_18_ column (250 × 10 mm, 5 µm), yielding **1** (5.5 mg, *t*_R_ 22.2 min) and **2** (1.5 mg, *t*_R_ 19.2 min).

### 4.5. Spectral Data of Compounds **1** and **2**

Enhypyrazinone A (**1**): yellow powder; UV (CH_3_OH/CHCl_3_ 1:1) λ_max_ (log ε) 315 (3.88) nm; IR (ATR) *υ*_max_ 3363.9, 2945.3, 2834.3, 1736.9, 1639.2, 1449.9, 1409.6, 1217.0, 1115.3, 1020.0 cm^−1^; ^1^H and ^13^C NMR (See Table 2); HRESIMS *m/z* 392.1736 [M + Na]^+^ (calcd. for C_24_H_23_N_3_ONa^+^, 392.1733).

Enhypyrazinone B (**2**): yellow powder; UV (CH_3_OH/CHCl_3_ 1:1) λ_max_ (log ε) 290 (3.89) nm; IR (ATR) *υ*_max_ 3341.0, 2944.4, 2832.5, 1737.6, 1641.7, 1449.1, 1229.0, 1116.2, 1022.9 cm^−1^; ^1^H and ^13^C NMR (See Table S1); HRESIMS *m/z* 392.1735 [M + Na]^+^ (calcd. for C_24_H_23_N_3_ONa^+^, 392.1733).

### 4.6. Media Recipe for Other Tested Conditions for the Growth of Enhygromyxa sp. WMMC2659

VY/2 medium contains 5.0 g Baker’s yeast cake, 0.5 mg cyanocobalamin per liter with 50% ASW. VY/4 medium is half-strength VY/2 per liter with 50% ASW. CY medium contains 3.0 g casitone, 1.0 g yeast extract per liter with 50% ASW. 1/3 CY and 1/6 CY media are one-third-strength and one-sixth-strength CY medium per liter with 50% ASW, respectively. R2A medium contains 0.5 g yeast extract, 0.5 g peptone, 0.5 g casamino acids, 0.5 g dextrose, 0.5 g soluble starch, 0.3 g sodium pyruvate, 0.3 g dipotassium phosphate, 0.05 g magnesium phosphate per liter with 50% ASW.

### 4.7. Antibacterial Assays

Compounds **1** and **2** were tested for antibacterial activity against *E. coli* (ATCC 25922), MRSA (ATCC 33591), and MSSA (ATCC 25913) in disk diffusion assays. Five microliters (10 mg/mL) of each compound was used for each disk, and only a 1 cm inhibition zone was observed against MSSA for both compounds **1** and **2**. MICs were further determined using a dilution antimicrobial susceptibility test for MSSA [31]. Compounds **1** and **2** were dissolved in DMSO, serially diluted to 10 concentrations (0.25–128 μg/mL), and tested in a 96-well plate. Vancomycin was used as the positive control against MSSA, which showed a MIC of 1 μg/mL. Compounds **1**, **2,** and the positive control were tested in duplicate. Eight untreated media controls were included on each plate. The plates were incubated at 33 °C for 18 h. The MIC was determined as the lowest concentration that inhibited visible growth of bacteria. 

## Figures and Tables

**Table 1:** Tested media conditions for *Enhygromyxa* sp. WMMC2659.

Media	*E. coli*	Dead *E. coli*	VY/2	VY/4	CY	1/3 CY	1/6 CY	R2A
Growth	Yes	Yes	No	No	No	No	No	No

**Table 2:** ^1^H and ^13^C NMR Data for Enhypyrazinone A (**1**) (600 MHz for ^1^H, 125 MHz for ^13^C, DMSO-*d_6_*).

Position	*δ_C_*, mult.	*δ*_H_ (*J* in Hz)	COSY	^1^H-^13^C HMBC ^a^	*δ*_N_^b^	^1^H–^15^N HMBC ^c^
1	156.3, C					
2					187.3	
3	137.3, C					
4	126.5, C					
5					323.2	
6	159.0, C					
7	25.6, CH_2_	4.14, s	9	3, 4, 8, 9, 16		2
8	111.1, C					
9	123.6, CH	7.22, s	7	8, 11, 16		10
10		11.0, s	9		132.0	
11	136.2, C					
12	111.6, CH	7.33, d (8.0)	13	14, 16		10
13	121.1, CH	7.05, t (8.0)		11, 15		
14	118.5, CH	6.97, t (8.0)	15	12, 16		
15	118.6, CH	7.65, d (8.0)	14	8, 11, 13, 16		
16	126.7, C					
17	123.1, CH	7.42, d (15.5)		4, 19		5
18	126.2, CH	7.24, d (15.5)		4, 19, 20, 24		
19	137.5, C					
20	126.3, CH	7.51, d (7.7)	21	22, 24		
21	128.7, CH	7.32, t (7.7)	20, 22	19, 23		
22	127.0, CH	7.20, t (7.7)	21, 23	20, 24		
23	128.7, CH	7.32, t (7.7)	22, 24	19, 21		
24	126.3, CH	7.51, d (7.7)	23	20, 22		
25	29.7, CH	3.30, m	26, 27	1, 6, 26, 27		5
26	20.2, CH_3_	1.19, d (6.8)	25	6, 25, 27		
27	20.2, CH_3_	1.19, d (6.8)	25	6, 25, 26		

^a^ HMBC correlations are from proton(s) to the indicated carbon. ^b^ The chemical shifts of ^15^N were determined by ^1^H–^15^N HMBC, and the chemical shifts of ^15^N were referenced to liquid NH_3_ (under pressure) at 25 °C by the NMR software TopSpin. ^c^ HMBC correlations are from proton(s) to the indicated nitrogen.

## Supplementary Materials

The following are available online at https://www.mdpi.com/1660-3397/17/12/698/s1, Figure S1: ^1^H NMR spectrum of enhypyrazinone A (**1**; 600 MHz, DMSO-*d*_6_), Figure S2: ^13^C NMR spectrum of enhypyrazinone A (**1**; 125 MHz, DMSO-*d*_6_), Figure S3: gCOSY spectrum of enhypyrazinone A (**1**; 600 MHz, DMSO-*d*_6_), Figure S4: gHSQC spectrum of enhypyrazinone A (**1**; 600 MHz, DMSO-*d*_6_), Figure S5: gHMBC spectrum of enhypyrazinone A (**1**; 600 MHz, DMSO-*d*_6_), Figure S6: ^1^H-^15^N HMBC spectrum of enhypyrazinone A (**1**; 600 MHz, DMSO-*d*_6_), Figure S7: Positive ion HRESIMS of enhypyrazinone A (**1**), Figure S8: ^1^H NMR spectrum of enhypyrazinone B (**2**; 500 MHz, CDCl_3_/CD_3_OD 1:1), Figure S9: ^13^C NMR spectrum of enhypyrazinone B (**2**; 125 MHz, CDCl_3_/CD_3_OD 1:1), Figure S10: gCOSY spectrum of enhypyrazinone B (**2**; 500 MHz, CDCl_3_/CD_3_OD 1:1), Figure S11: gHSQC spectrum of enhypyrazinone B (**2**; 500 MHz, CDCl_3_/CD_3_OD 1:1), Figure S12: gHMBC spectrum of enhypyrazinone B (**2**; 500 MHz, CDCl_3_/CD_3_OD 1:1), Figure S13: ^1^H-^15^N HMBC spectrum of enhypyrazinone B (**2**; 500 MHz, CDCl_3_/CD_3_OD 1:1), Figure S14: ^1^H NMR spectrum of enhypyrazinone B (**2**; 500 MHz, DMSO-*d*_6_), Figure S15: ^13^C NMR spectrum of enhypyrazinone B (**2**; 125 MHz, DMSO-*d*_6_), Figure S16: gCOSY spectrum of enhypyrazinone B (**2**; 500 MHz, DMSO-*d*_6_), Figure S17: gHSQC spectrum of enhypyrazinone B (**2**; 500 MHz, DMSO-*d*_6_), Figure S18: gHMBC spectrum of enhypyrazinone B (**2**; 500 MHz, DMSO-*d*_6_), Figure S19: ^1^H-^15^N HMBC spectrum of enhypyrazinone B (**2**; 500 MHz, DMSO-*d*_6_), Figure S20: Positive ion HRESIMS of enhypyrazinone B (**2**), Table S1: ^1^H and ^13^C NMR data for enhypyrazinone B (**2**) (500 MHz for ^1^H, 125 MHz for ^13^C, CDCl_3_/CD_3_OD 1:1).
