# Supplementary material for: Enhypyrazinones A and B, Pyrazinone Natural Products from a Marine-Derived Myxobacterium Enhygromyxa sp
Source: Mar Drugs. 2019 Dec 12;17(12):698. doi: 10.3390/md17120698 (PMC6950740; doi:10.3390/md17120698)
Supplement: Supplementary file 1 [file marinedrugs-17-00698-s001.pdf]

Supplementary

Enhypyrazinones A and B, Pyrazinone Natural Products from a Marine-derived Myxobacterium *Enhygromyxa* sp.

Fan Zhang,[1] Doug R. Braun,[1] Scott R. Rajski,[1] Don DeMaria,[2] and Tim S. Bugni [1,*]

[1]Pharmaceutical Sciences Division, University of Wisconsin–Madison, Madison, Wisconsin 53705, United States

[2]Sea Samples, 369 Westshore Drive, Summerland Key, FL 33042, United States

* To whom correspondence should be addressed. Tel.: 1-608-263-2519. E-mail: tim.bugni@wisc.edu.

Supporting Information Table of Contents

Contents **Page**

1) **Figure S1.** ^{1}H NMR spectrum of enhypyrazinone A (**1**; 600 MHz, DMSO-d_6)................. 3
2) **Figure S2.** ^{13}C NMR spectrum of enhypyrazinone A (**1**; 125 MHz, DMSO-d_6)................ 4
3) **Figure S3.** gCOSY spectrum of enhypyrazinone A (**1**; 600 MHz, DMSO-d_6)................. 5
4) **Figure S4.** gHSQC spectrum of enhypyrazinone A (**1**; 600 MHz, DMSO-d_6).................. 6
5) **Figure S5.** gHMBC spectrum of enhypyrazinone A (**1**; 600 MHz, DMSO-d_6)................. 7
6) **Figure S6.** ^{1}H-^{15}N HMBC spectrum of enhypyrazinone A (**1**; 600 MHz, DMSO-d_6)........ 8
7) **Figure S7.** Positive ion HRESIMS of enhypyrazinone A (**1**)... 9
8) **Figure S8.** ^{1}H NMR spectrum of enhypyrazinone B (**2**; 500 MHz, $CDCl_3/CD_3OD$ 1:1)... 10
9) **Figure S9.** ^{13}C NMR spectrum of enhypyrazinone B (**2**; 125 MHz, $CDCl_3/CD_3OD$ 1:1)...11
10) **Figure S10.** gCOSY spectrum of enhypyrazinone B (**2**; 500 MHz, $CDCl_3/CD_3OD$ 1:1).12
11) **Figure S11.** gHSQC spectrum of enhypyrazinone B (**2**; 500 MHz, $CDCl_3/CD_3OD$ 1:1).. 13
12) **Figure S12.** gHMBC spectrum of enhypyrazinone B (**2**; 500 MHz, $CDCl_3/CD_3OD$ 1:1). 14
13) **Figure S13.** ^{1}H-^{15}N HMBC Spectrum of enhypyrazinone B (**2**; 500 MHz, $CDCl_3/CD_3OD$ 1:1).. 15
14**)** **Figure S14.** ^{1}H NMR spectrum of enhypyrazinone B (**2**; 500 MHz, DMSO-d_6)................16
15) **Figure S15.** ^{13}C NMR spectrum of enhypyrazinone B (**2**; 125 MHz, DMSO-d_6)............. 17
16) **Figure S16.** gCOSY spectrum of enhypyrazinone B (**2**; 500 MHz, DMSO-d_6)................ 18
17) **Figure S17.** gHSQC spectrum of enhypyrazinone B (**2**; 500 MHz, DMSO-d_6)................ 19
18) **Figure S18.** gHMBC spectrum of enhypyrazinone B (**2**; 500 MHz, DMSO-d_6)................ 20
19) **Figure S19.** ^{1}H-^{15}N HMBC spectrum of enhypyrazinone B (**2**; 500 MHz, DMSO-d_6)...... 21
20) **Figure S20.** Positive ion HRESIMS of enhypyrazinone B (**2**)... 22
21) **Table S1.** ^{1}H and ^{13}C NMR data for enhypyrazinone B (**2**) (500 MHz for ^{1}H, 125 MHz for ^{13}C, $CDCl_3/CD_3OD$ 1:1) 23

Figure S1. ^{1}H NMR spectrum of enhypyrazinone A (**1**; 600 MHz, DMSO-d_6)

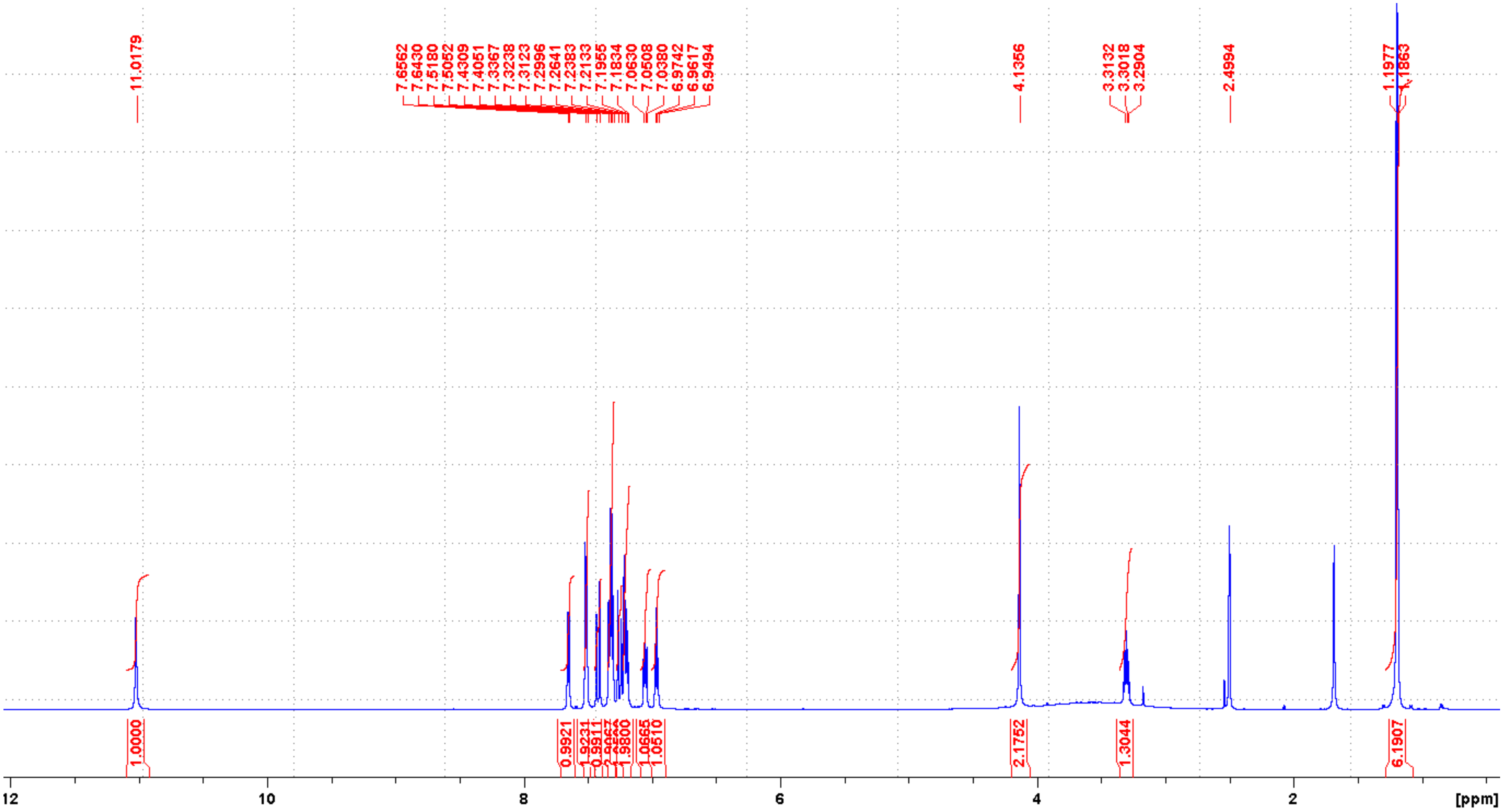

Figure S2. ^{13}C NMR spectrum of enhypyrazinone A (**1**; 125 MHz, DMSO-d_6)

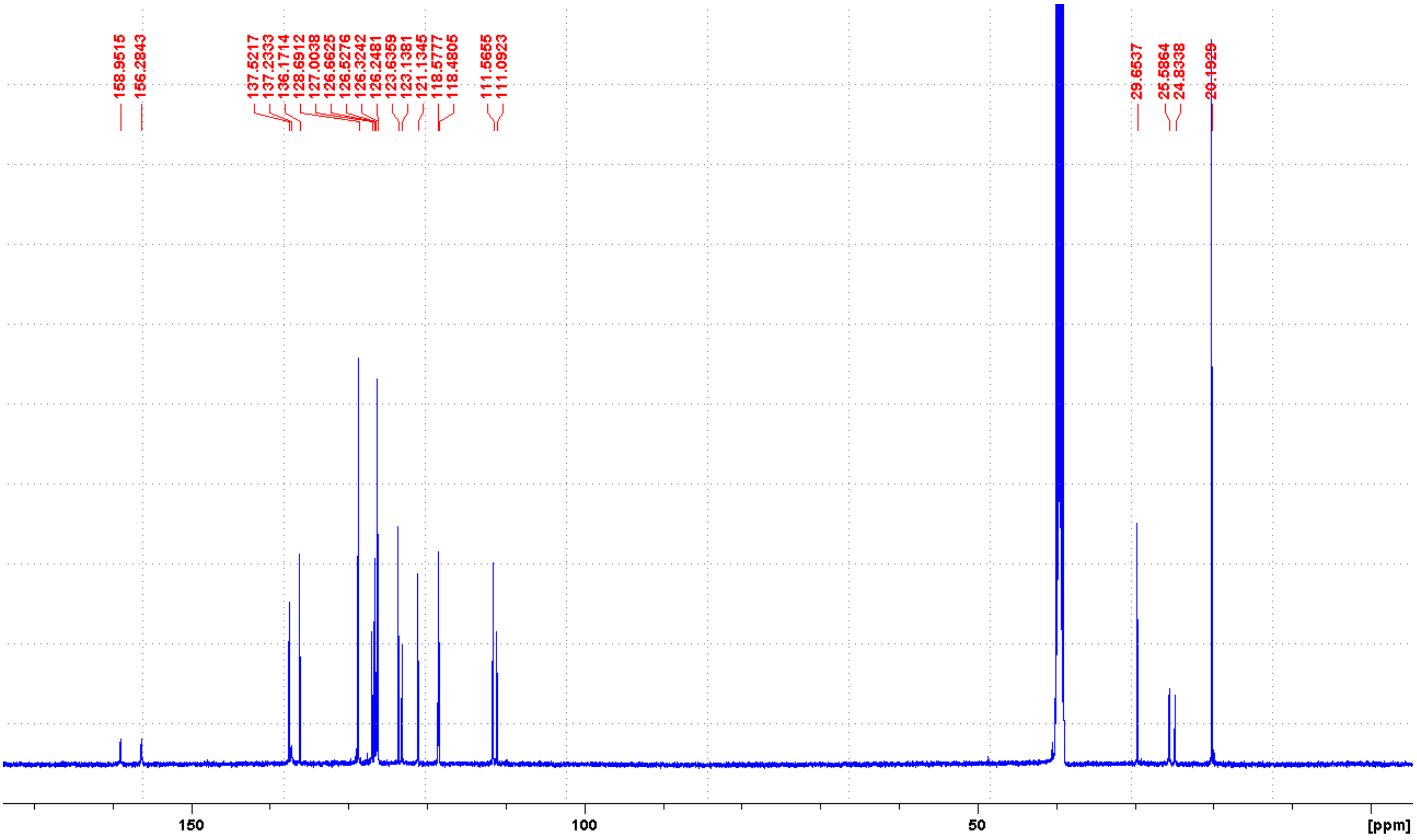

Figure S3. gCOSY spectrum of enhypyrazinone A (**1**; 600 MHz, DMSO-d_6)

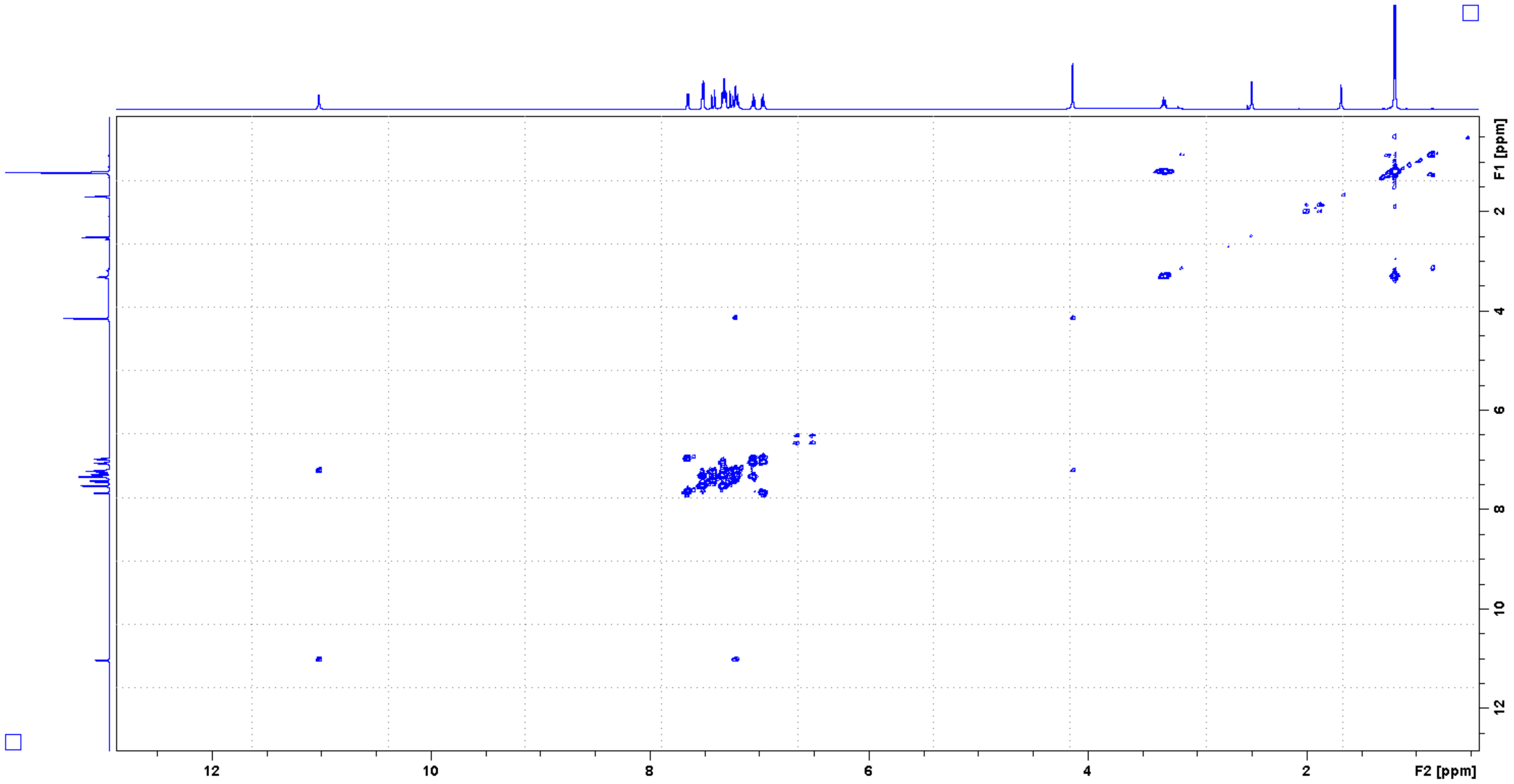

Figure S4. gHSQC spectrum of enhypyrazinone A (**1**; 600 MHz, DMSO-d_6)

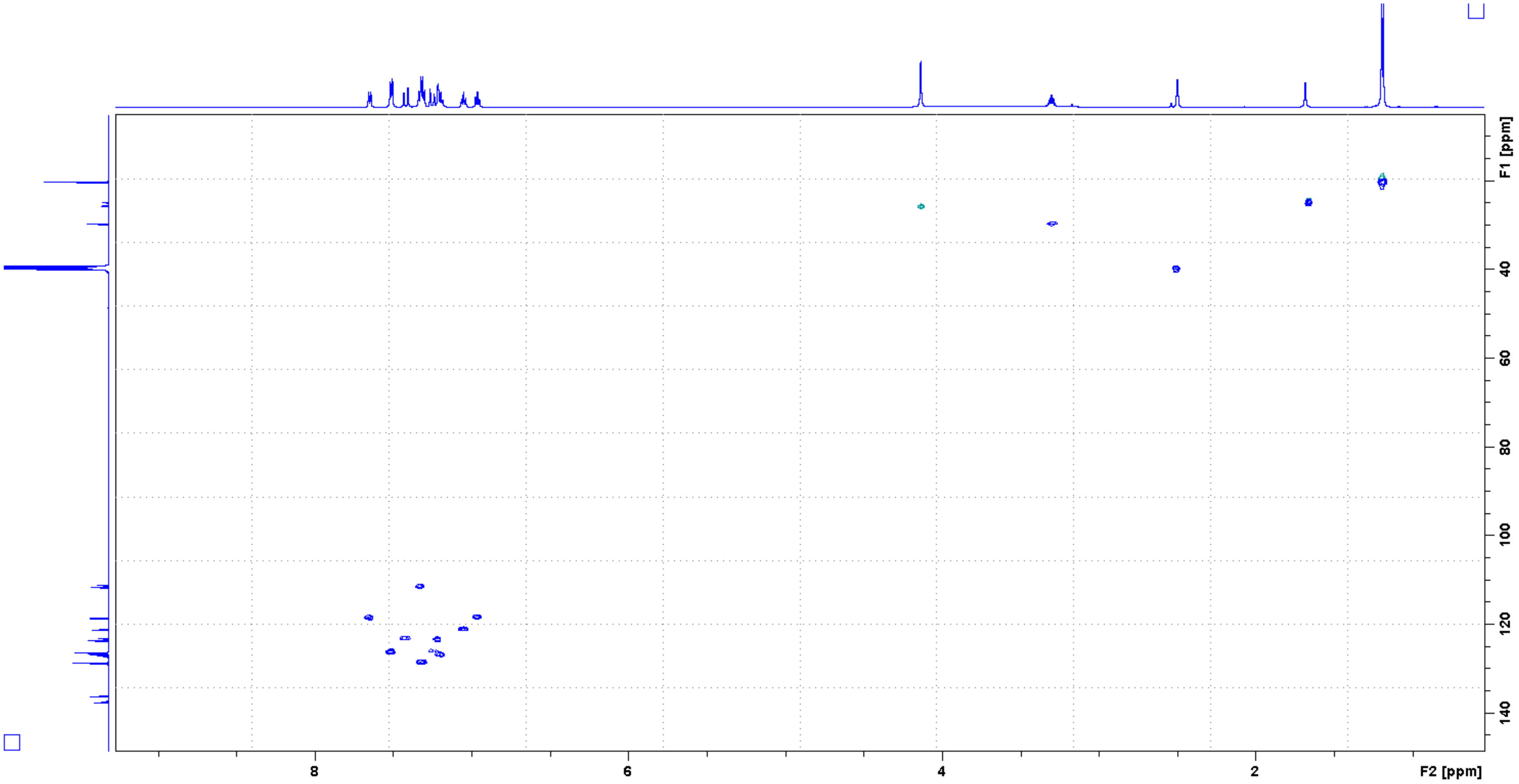

Figure S5. gHMBC spectrum of enhypyrazinone A (**1**; 600 MHz, DMSO-d_6)

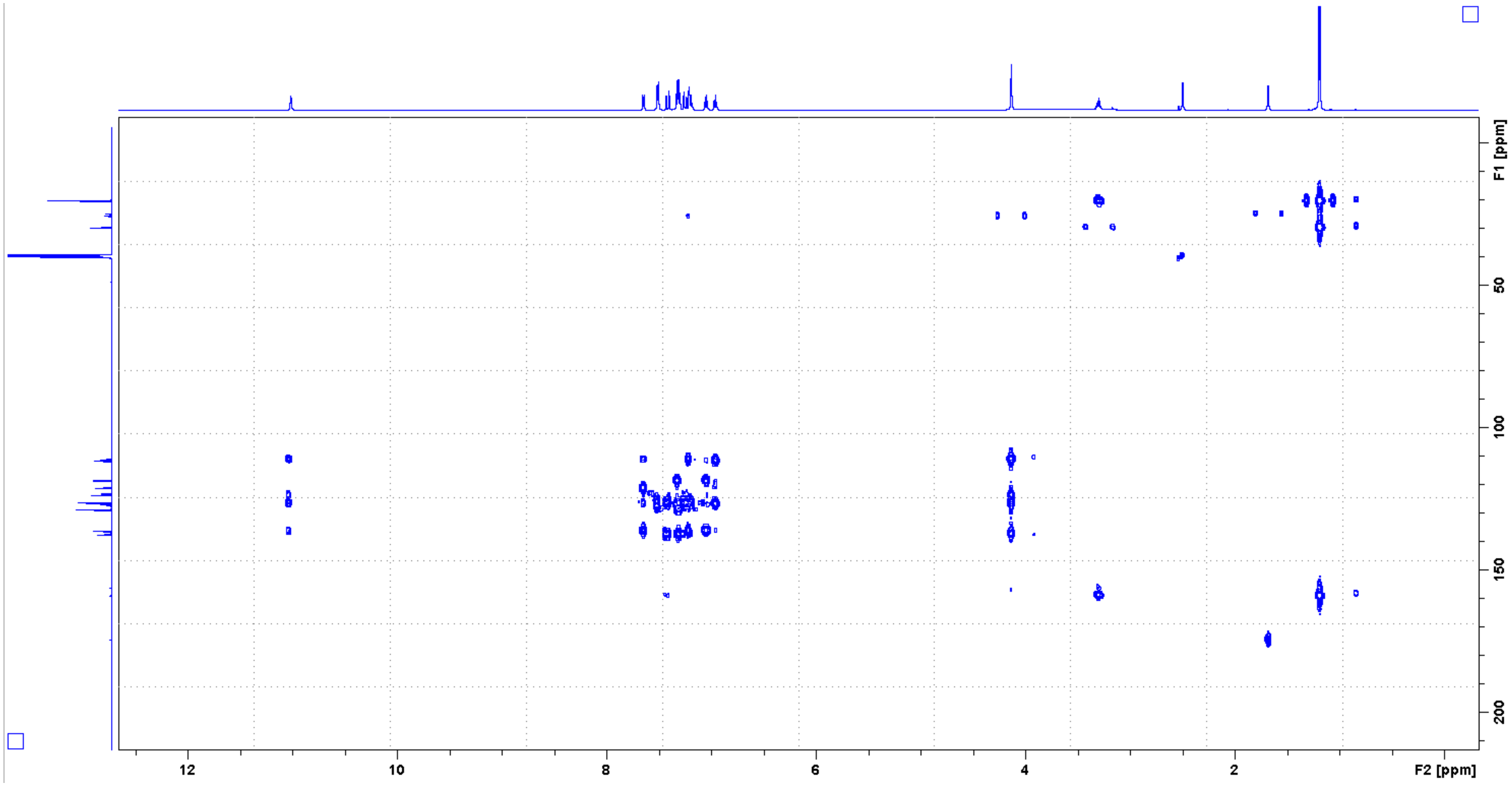

Figure S6. ^{1}H-^{15}N HMBC spectrum of enhypyrazinone A (**1**; 600 MHz, DMSO-d_6)

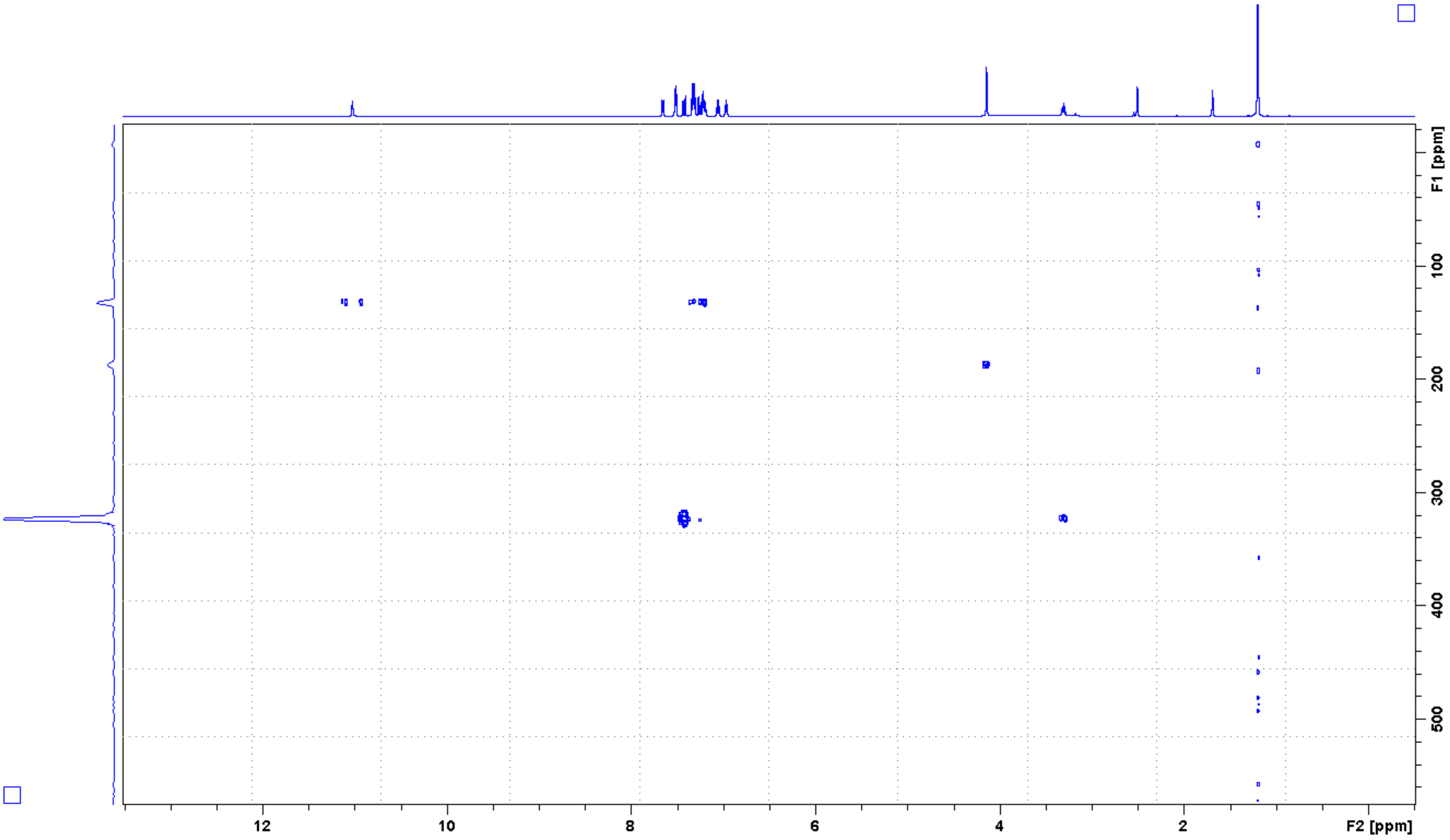

Figure S7. Positive ion HRESIMS of enhypyrazinone A (**1**)

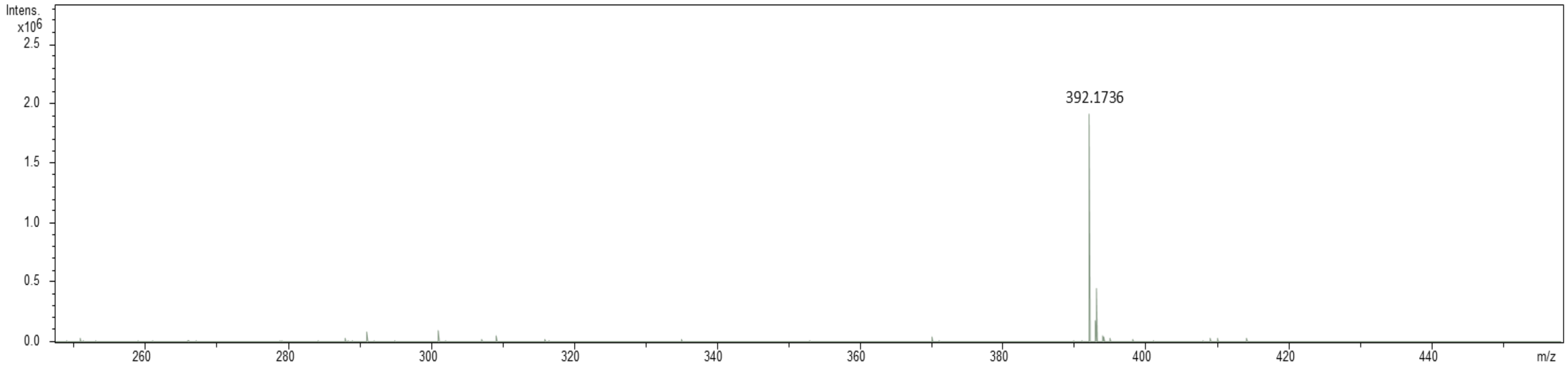

Figure S8. ^{1}H NMR spectrum of enhypyrazinone B (**2**; 500 MHz, $CDCl_3/CD_3OD$ 1:1)

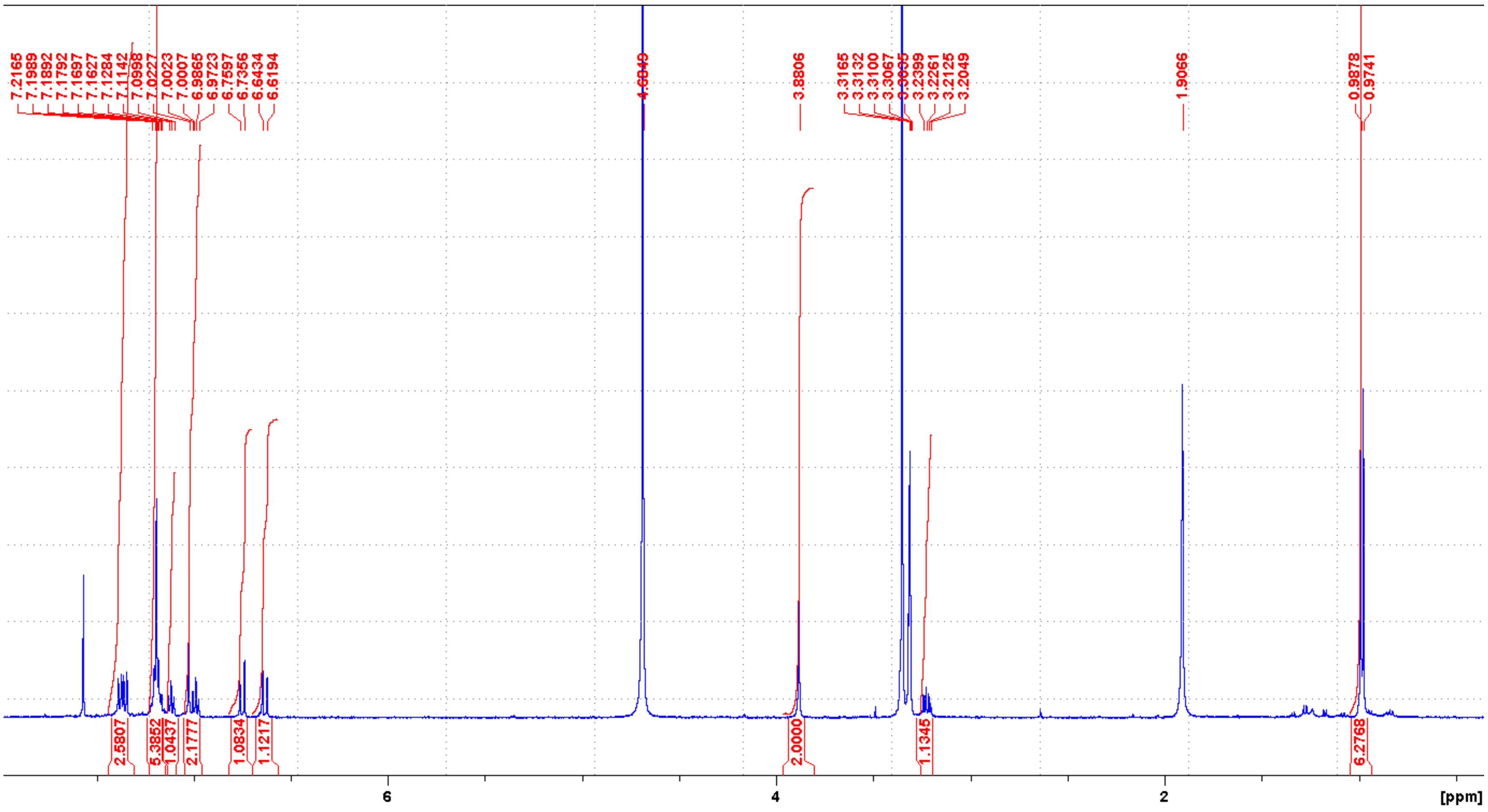

Figure S9. ^{13}C NMR spectrum of enhypyrazinone B (**2**; 125 MHz, $CDCl_3/CD_3OD$ 1:1)

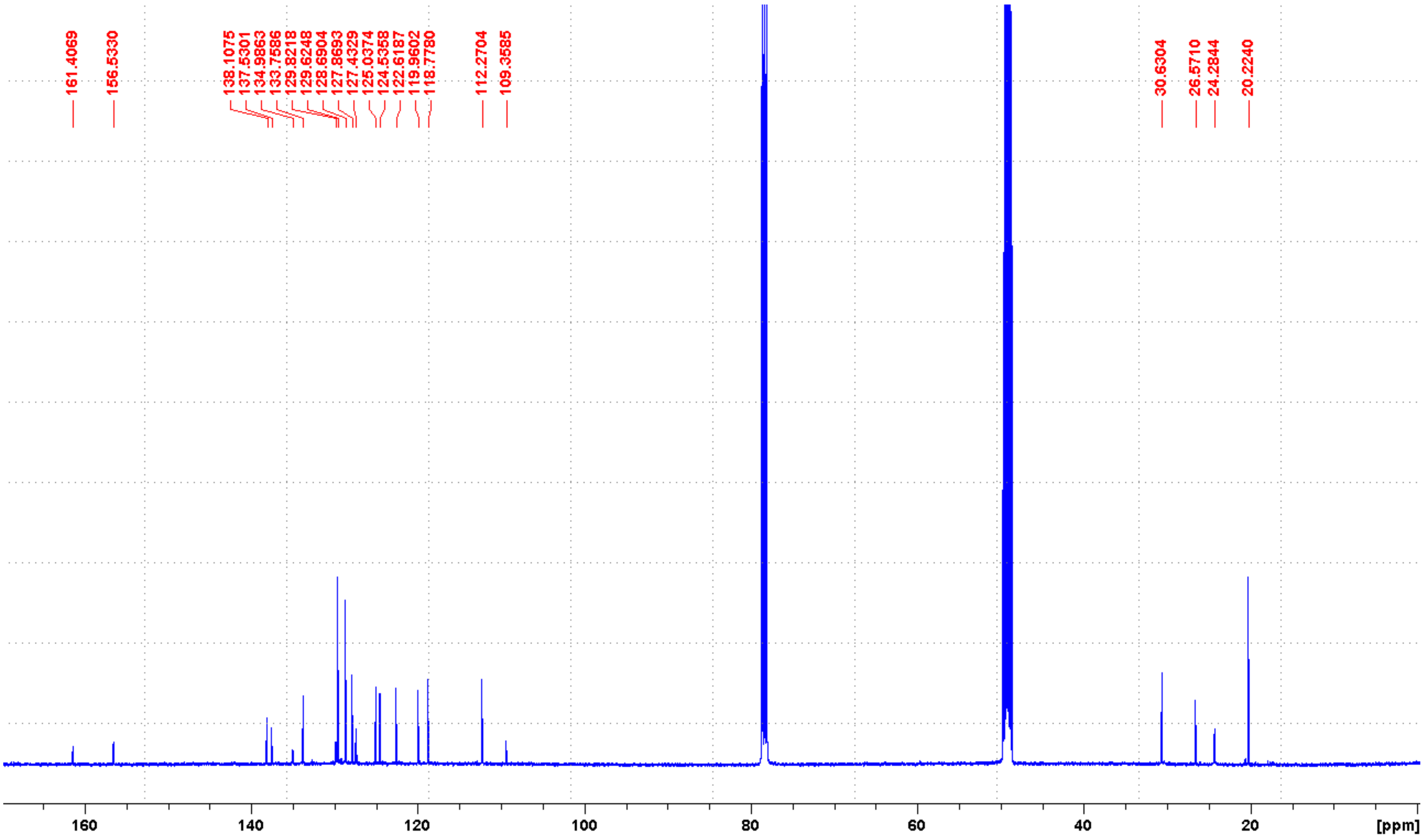

Figure S10. gCOSY spectrum of enhypyrazinone B (**2**; 500 MHz, $CDCl_3/CD_3OD$ 1:1)

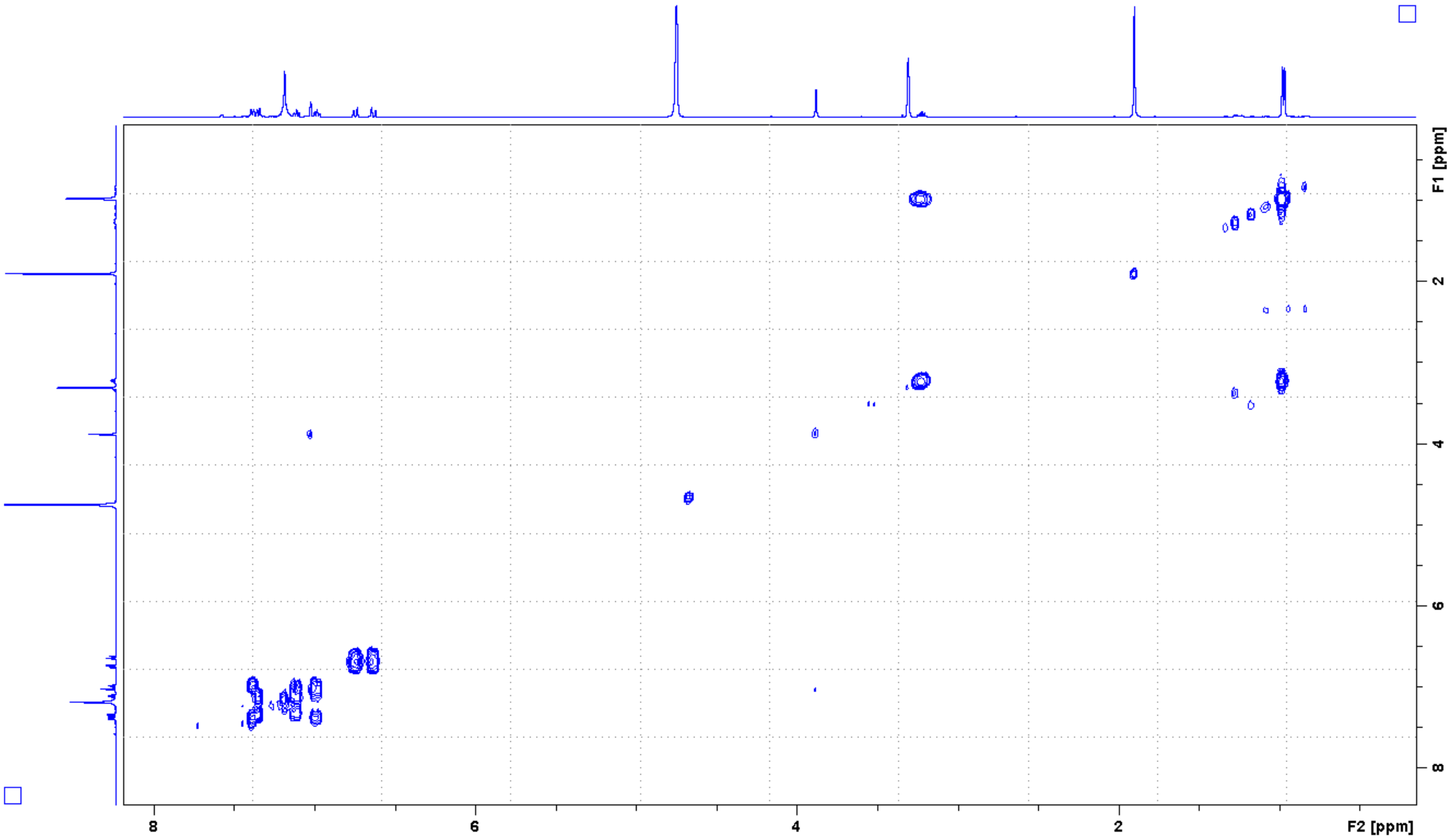

Figure S11. gHSQC spectrum of enhypyrazinone B (**2**; 500 MHz, $CDCl_3/CD_3OD$ 1:1)

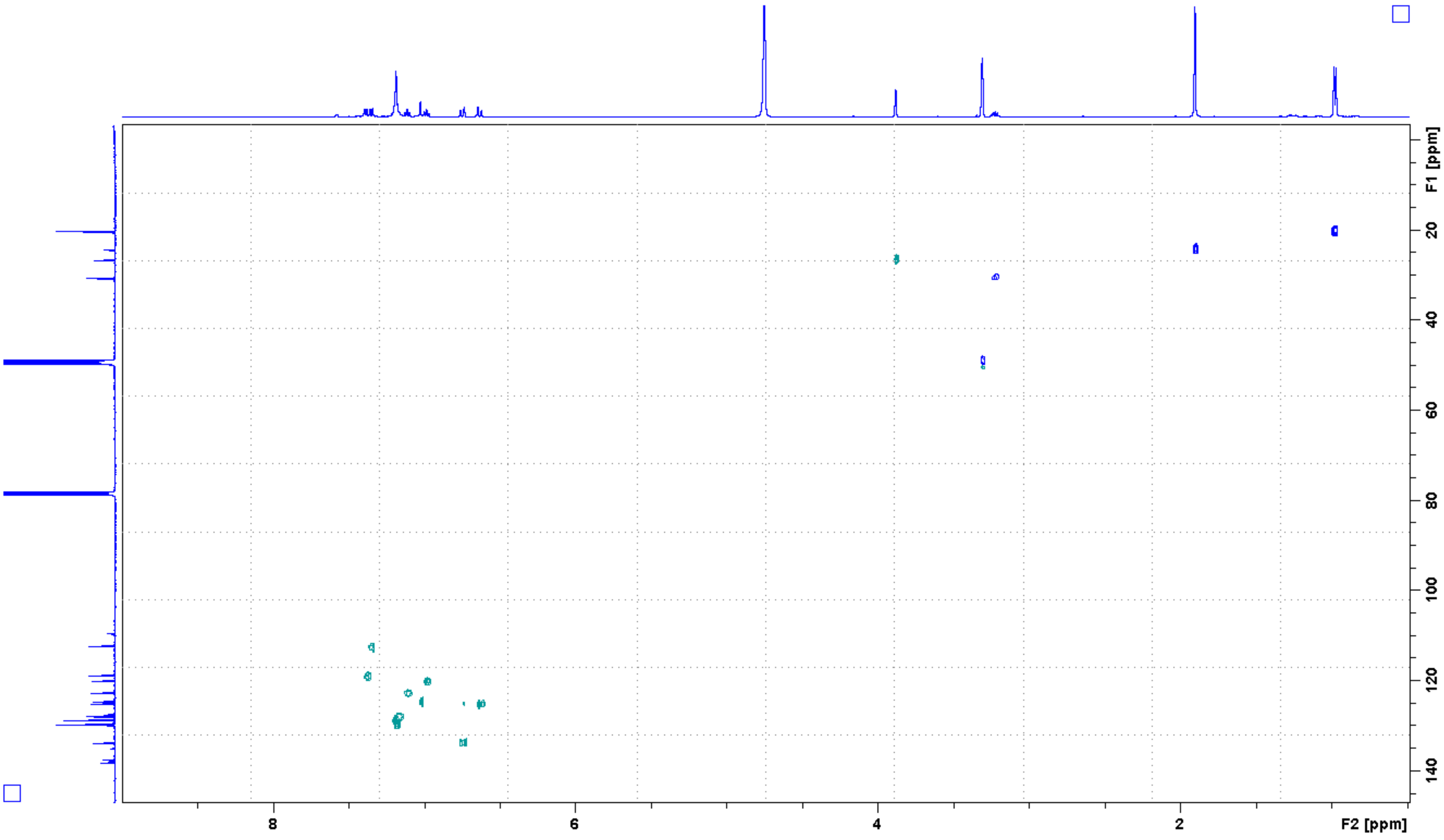

Figure S12. gHMBC spectrum of enhypyrazinone B (**2**; 500 MHz, $CDCl_3/CD_3OD$ 1:1)

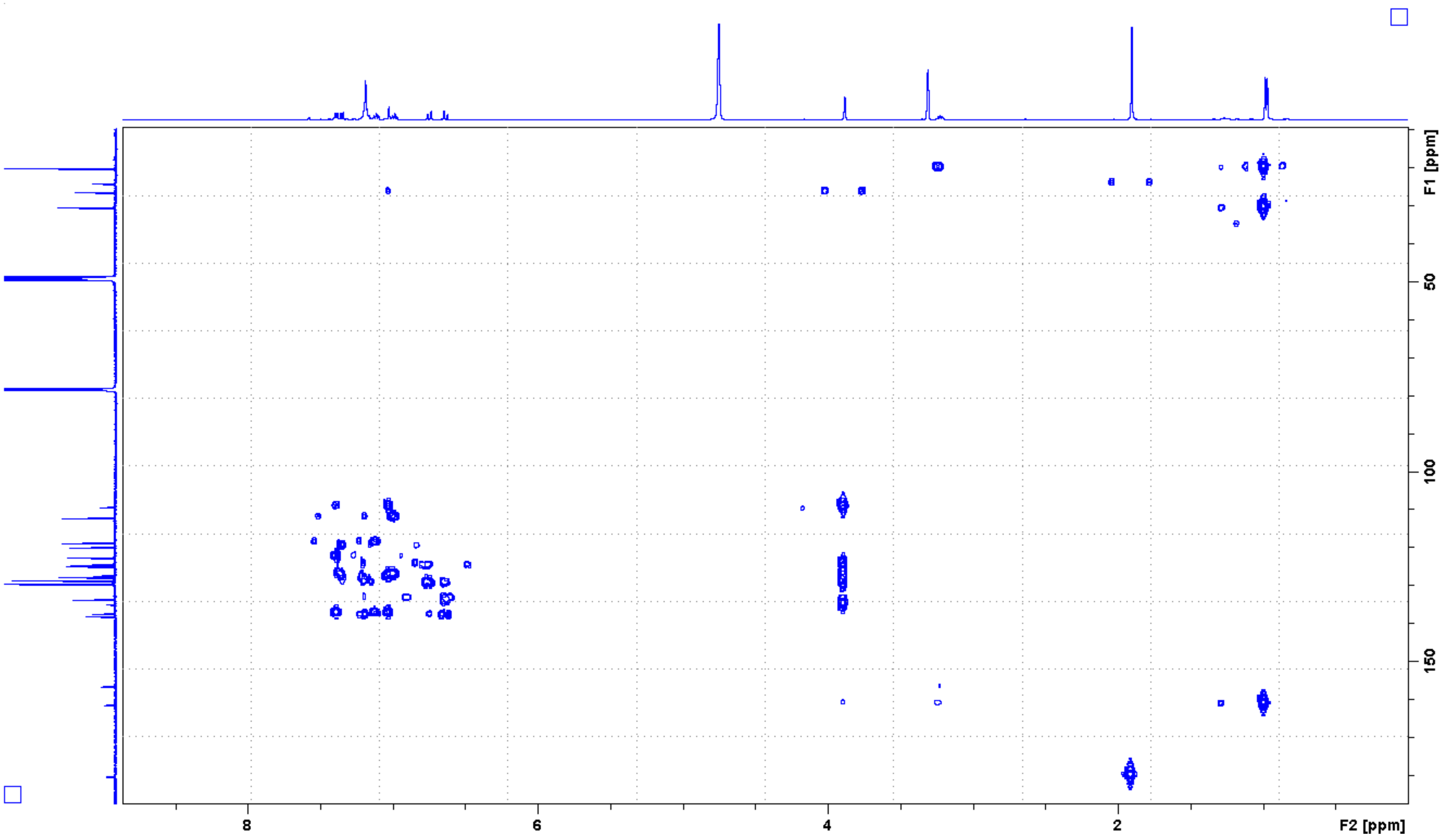

Figure S13. ^{1}H-^{15}N HMBC Spectrum of enhypyrazinone B (**2**; 500 MHz, $CDCl_3$/CD_3OD 1:1)

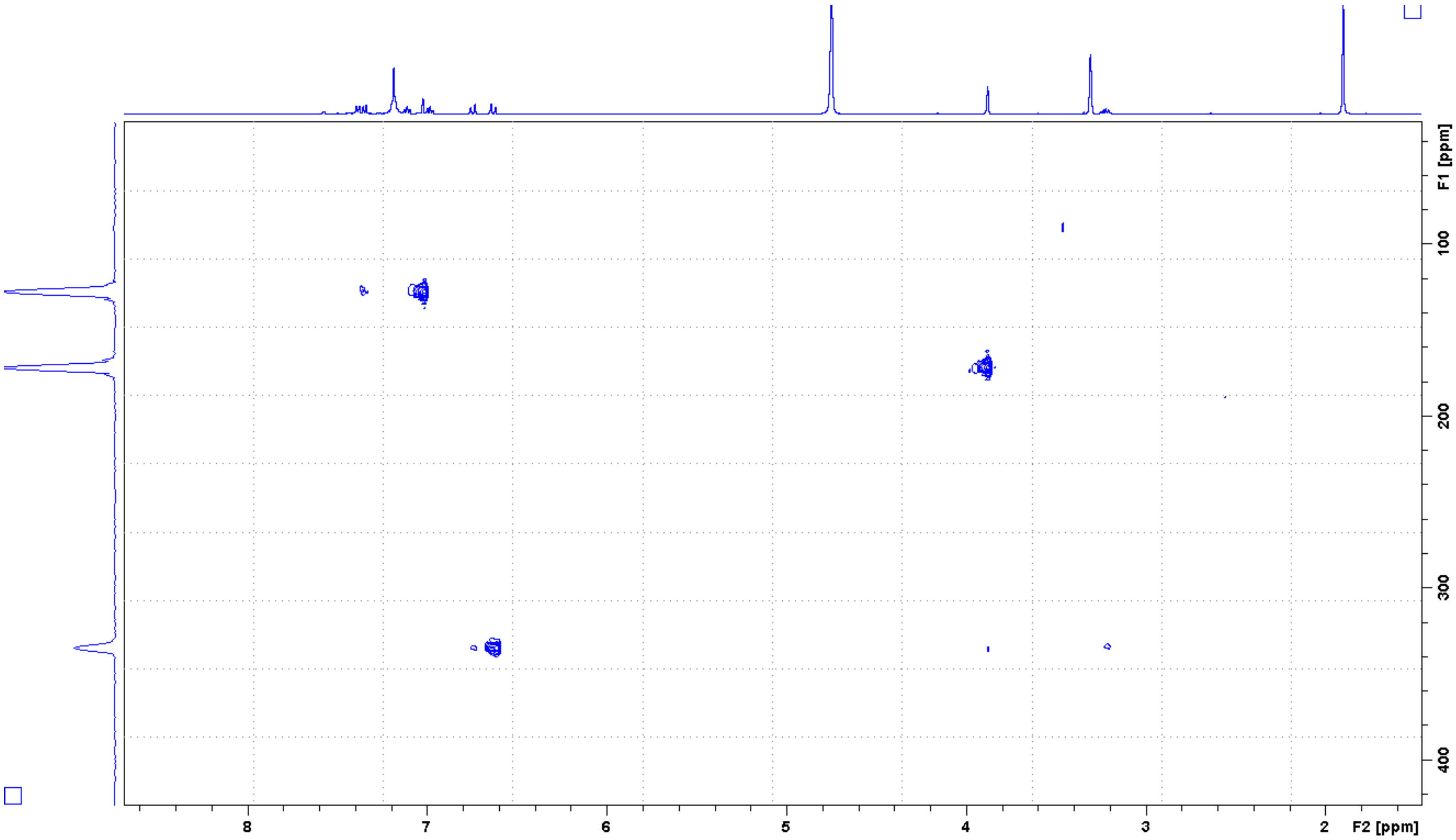

Figure S14. ^{1}H NMR spectrum of enhypyrazinone B (**2**; 500 MHz, DMSO-d_6)

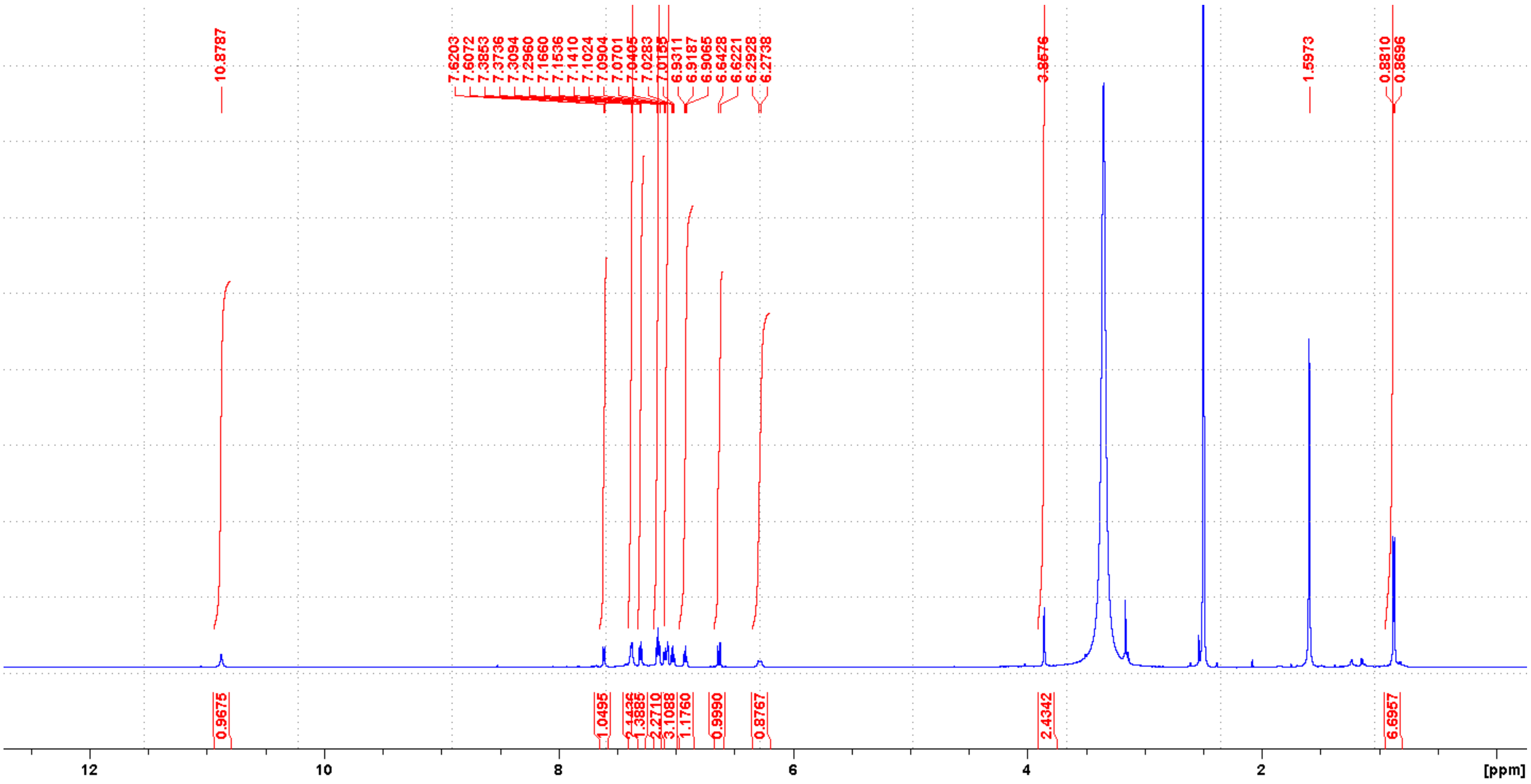

Figure S15. ^{13}C NMR spectrum of enhypyrazinone B (**2**; 125 MHz, DMSO-d_6)

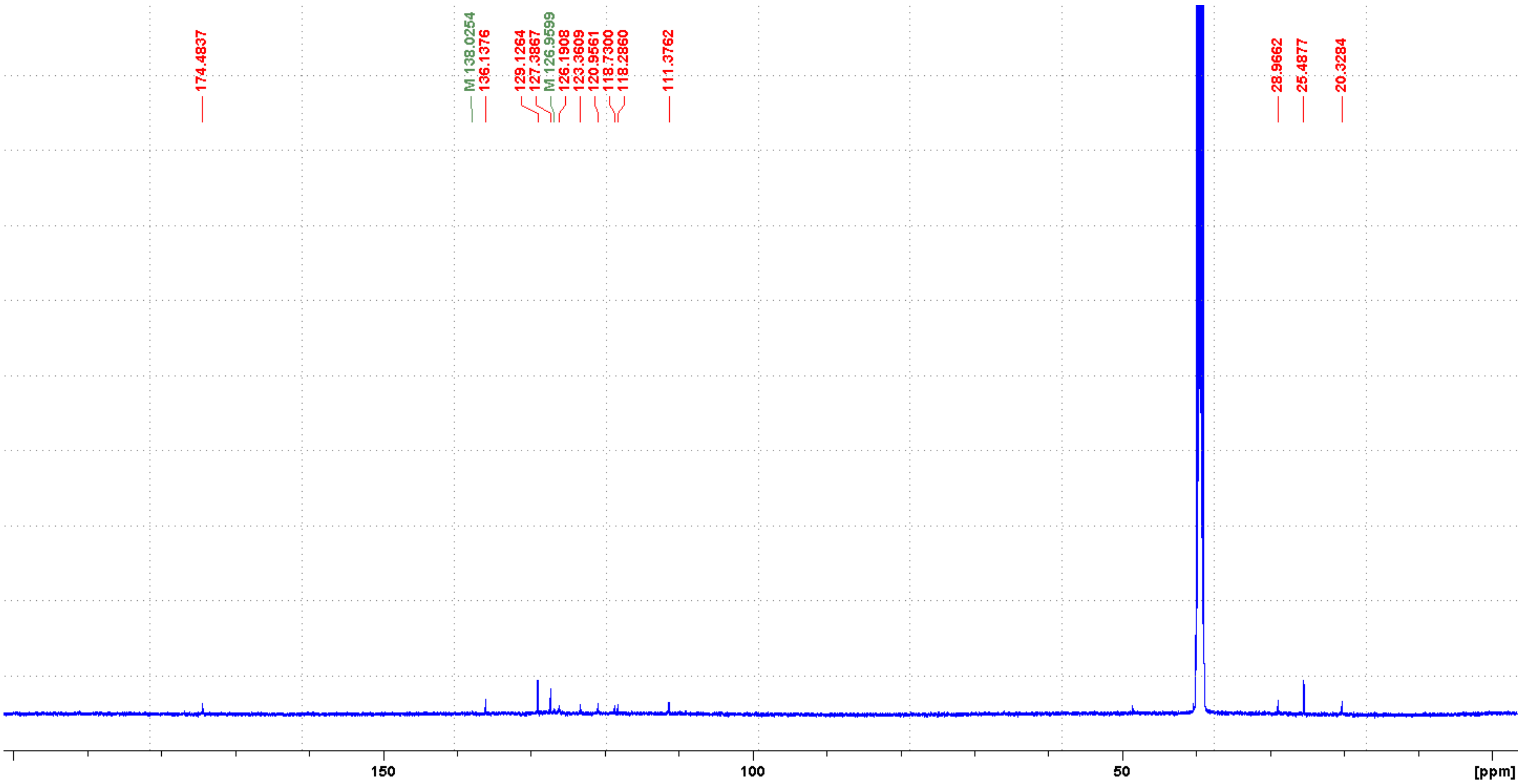

Figure S16. gCOSY spectrum of enhypyrazinone B (**2**; 500 MHz, DMSO-d_6)

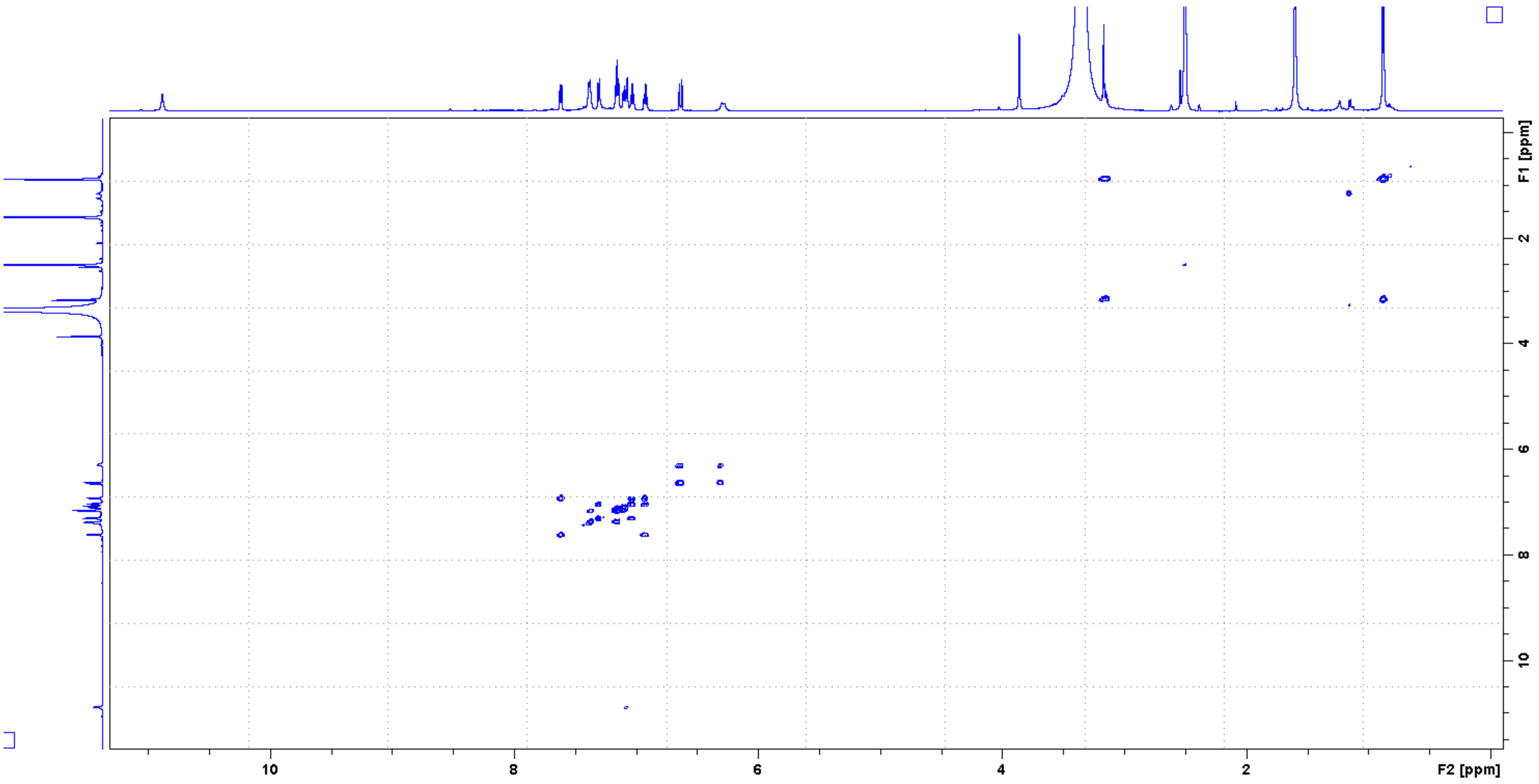

Figure S17. gHSQC spectrum of enhypyrazinone B (**2**; 500 MHz, DMSO-d_6)

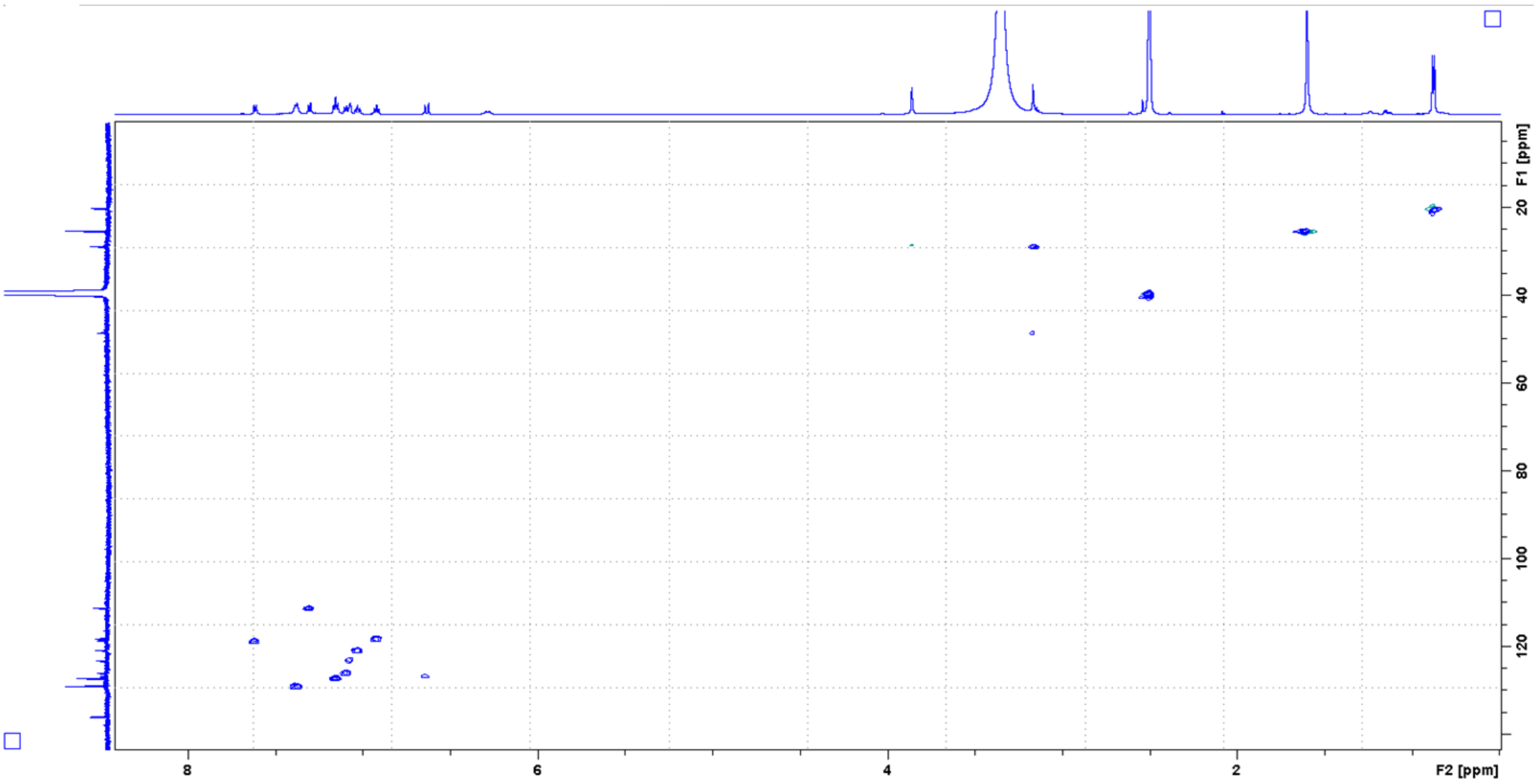

Figure S18. gHMBC spectrum of enhypyrazinone B (**2**; 500 MHz, DMSO-d_6)

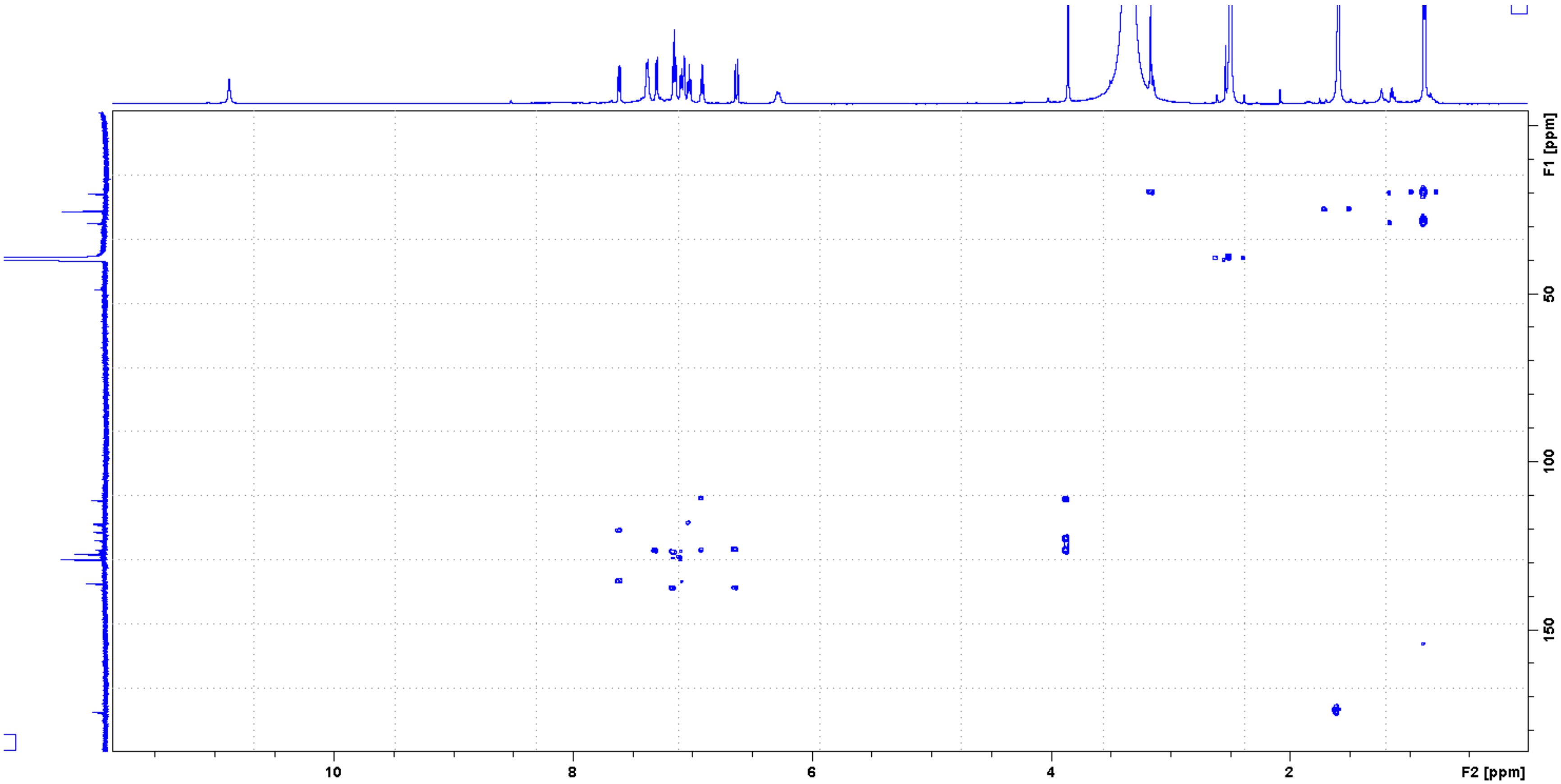

Figure S19. ^{1}H-^{15}N HMBC spectrum of enhypyrazinone B (**2**; 500 MHz, DMSO-d_6)

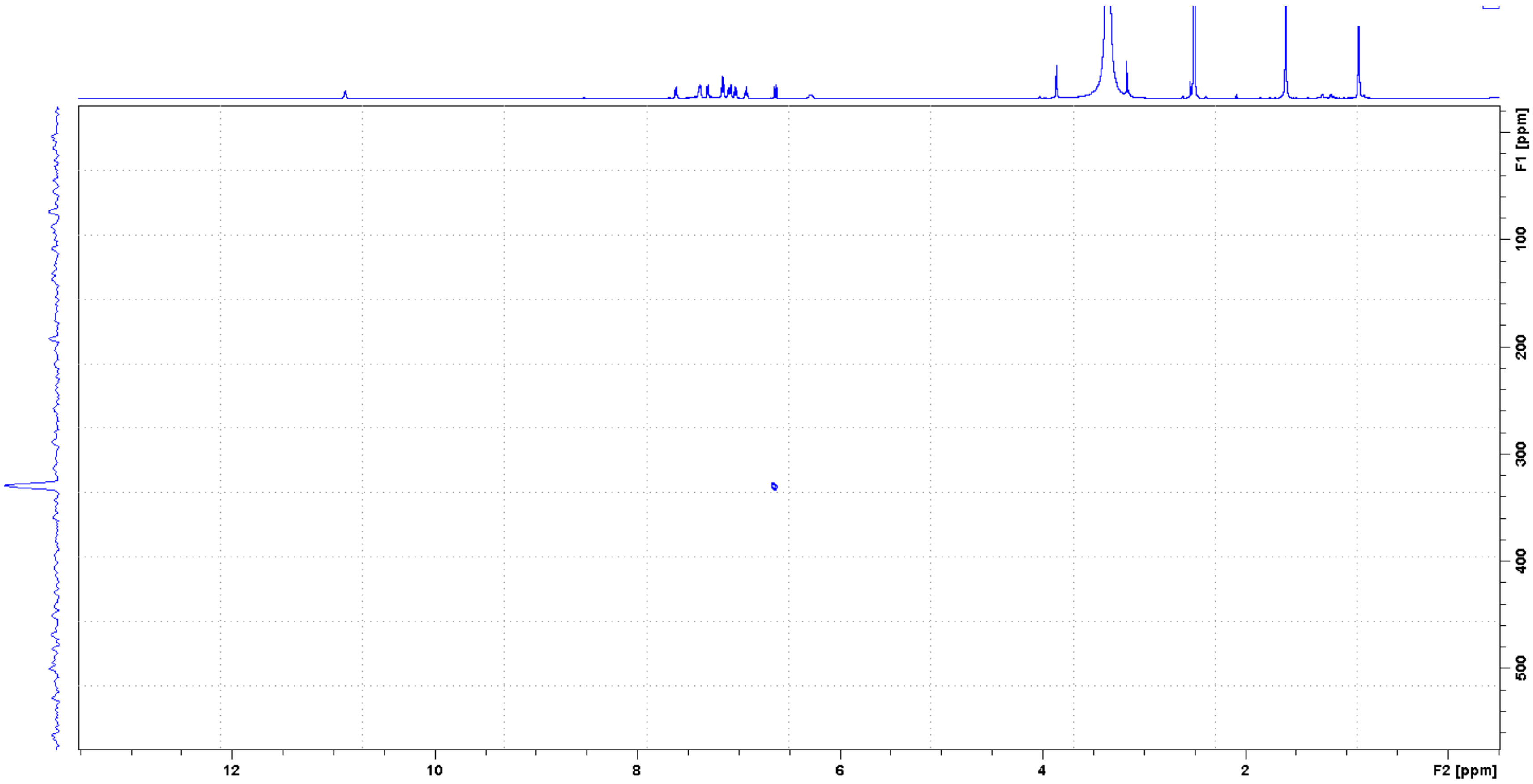

Figure S20. Positive ion HRESIMS of enhypyrazinone B (**2**)

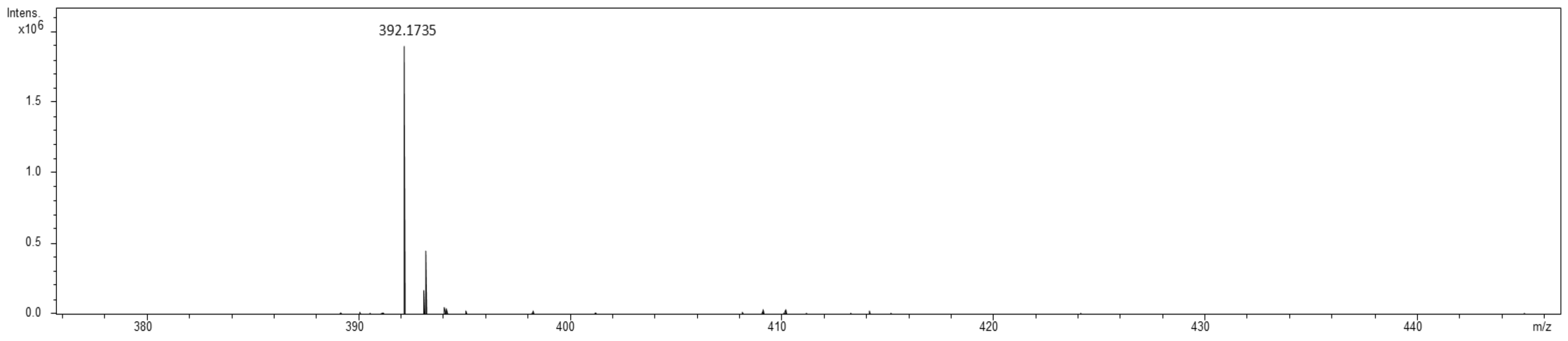

Table 1. ^{1}H and ^{13}C NMR data for enhypyrazinone B (**2**) (500 MHz for ^{1}H, 125 MHz for ^{13}C, $CDCl_3/CD_3OD$ 1:1).

Positon	δ_C, mult.	δ_H (*J* in Hz)	^{1}H-^{1}H COSY	^{1}H-^{13}C HMBC[a]	δ_N[b]	^{1}H-^{15}N HMBC[c]
1	156.5, qC					
2					172.1	
3	135.0, qC					
4	129.8, qC					
5					335.1	
6	161.4, qC					
7	26.6, CH_2	3.88, s	9	3, 4, 8, 9, 16		2
8	109.4, qC					
9	124.5, CH	7.03, s	7	8, 11, 16		10
10					127.7	
11	137.5, qC					
12	112.3, CH	7.35, d (8.0)	13	14, 16		10 (weak)
13	122.6, CH	7.11, t (8.0)		11, 15		
14	120.0, CH	6.99, t (8.0)	15	12, 15		
15	118.8, CH	7.39, d (8.0)	14	8, 11, 13		
16	127.4, qC					
17	125.0, CH	6.64, d (12.0)	18	3, 4, 18, 19		5
18	133.8, CH	6.75, d (12.0)	17	4, 17, 19		
19	138.1, qC					
20	129.6, CH	7.19, m		18, 19, 21		
21	128.7, CH	7.22, m		22		
22	127.9, CH	7.16, m		20, 24		
23	128.7, CH	7.22, m		24		
24	129.6, CH	7.19, m		18, 19, 23		
25	30.6, CH	3.22, m	26, 27	6, 26, 27		5 (weak)
26	20.2, CH_3	0.98, d (6.8)	25	6, 25, 27		
27	20.2, CH_3	0.98, d (6.8)	25	6, 25, 26		

[a]HMBC correlations are from proton(s) to the indicated carbon.
[b]The chemical shifts of ^{15}N were determined by ^{1}H-^{15}N-HMBC.
[c]HMBC correlations are from proton(s) to the indicated nitrogen
